# Molecular signatures of mesenchymal stem cell-derived extracellular vesicle-mediated tissue repair

**DOI:** 10.1186/s13287-015-0214-y

**Published:** 2015-11-11

**Authors:** Takeshi Katsuda, Takahiro Ochiya

**Affiliations:** Division of Molecular and Cellular Medicine, National Cancer Center Research Institute, 5-1-1 Tsukiji, Chuo-ku, Tokyo 104-0045 Japan

## Abstract

Extracellular vesicles (EVs) play important roles in intercellular communications via their content molecules, and mimic, at least in part, the roles that are played by their originating cells. Consistent with this notion, an increasing number of reports have suggested that EVs derived from mesenchymal stem cells (MSCs), which are therapeutically beneficial to a wide range of diseases, can serve as drugs to treat multiple diseases. EVs contain a variety of molecules, including proteins, microRNAs, and mRNAs, and are associated with biological processes in a content molecule-dependent manner. In this article, we review the latest reports regarding the therapeutic potential of MSC-EVs by focusing on the underlying molecular mechanisms of their effects. Specifically, we feature the effects of MSC-EVs in terms of their content molecules and of the tissue recovery processes endowed by these molecules.

## Introduction

Interest in extracellular vesicles (EVs), lipid-bilayered vesicles that are secreted by various types of cells, as novel carriers for drug delivery systems has increased. In a broad sense, EVs include all types of vesicles that exist in the extracellular space. In particular, 50–200 nm EVs, usually termed exosomes [1] and shedding microvesicles [2], are the primary focus for many researchers owing to their biological significance. Thus, in this article, we use the term “EV” to refer to a 50–200 nm vesicle. Although the functions of EVs are variable, their functions often reflect the phenotypes of their originating cells.

Extracellular vesicles derived from mesenchymal stem cells (MSC-EVs) have therapeutic benefits against multiple diseases [3–5]. Mesenchymal stem cells (MSCs) are stem cells that reside in adult tissues and that assist in injury recovery. These cells have thus attracted much attention as a cell source for regenerative medicine. More recently, increasing numbers of reports have indicated that MSC-EVs show therapeutic effects similar to those which can be achieved by the originating MSCs themselves. These findings imply a novel therapeutic strategy using MSC-EVs as drugs for future regenerative medicine.

**Fig. 1:**
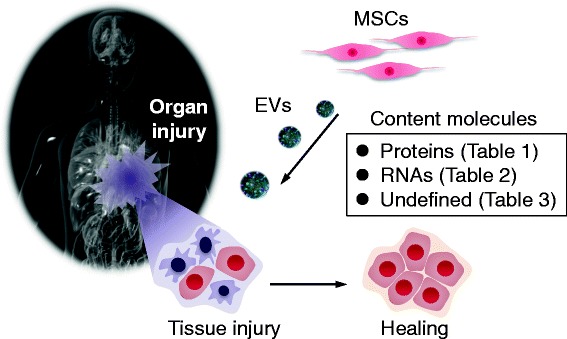
Schematic representation of therapeutic effects mediated by MSC-EVs. MSC-EVs can support the recovery of injured organs, and this supportive effect is dependent on EV content molecules. In this article, content molecule-dependent therapeutic effects are classified into three categories: protein-mediated effects, RNA-mediated effects, and undefined molecule-mediated effects (summarized in Tables 1, 2 and 3, respectively). *EV* extracellular vesicle, *MSC* mesenchymal stem cell

In this article, we review the latest reports regarding the therapeutic potential of MSC-EVs from the point of view of their content molecules. To this end, we classify their content molecules into three groups: proteins, RNAs, and undefined molecules. The possible molecular mechanisms underlying MSC-EV-mediated therapeutic effects are discussed (Fig. 1). We also discuss the further therapeutic potential of MSC-EVs in terms of the findings obtained from comprehensive analyses of molecular components of MSC-EVs; for example, RNA sequencing and proteomic analyses of MSC-EVs.

## Protein-mediated therapeutic effects

Evidence for biological functions of EV proteins was first provided by immunology studies in the late 1990s [6–10]. Thereafter, in the 2000s, the involvement of EV proteins was further described in other fields including cancer biology [11–13] and neuroscience [14–17]. Evidence for the therapeutic effects of MSC-EV proteins was not provided until after 2010 (Table 1).

**Table 1:** MSC-EV protein-mediated therapeutic effects

Species/MSC origin	Injured organ/experimental model	Responsible protein	Possible mechanisms for the therapeutic effect	Reference
Human/ESCs	Heart/mouse model of MIR injury (ex vivo)	ND	Predicted to have significant biological effects on cardiac tissue injury and repair, but the responsible molecules were not specifically determined	[18]
Human/fetus	Heart/mouse model of MIR injury (ex vivo)	ND	Attenuated TGFβ signaling and reduced apoptosis	[19]
Human/ESCs	Heart/mouse model of MIR (in vivo)	ND	Restoration of bioenergetics, reduction of oxidative stress and activation of pro-survival signaling, followed by enhancement of cardiac function and geometry after MIR injury	[20]
			Increased ATP and NADH, decreased oxidative stress, increased phosphorylated Akt and phosphorylated GSK-3β levels, and reduced phosphorylated c-JNK levels	
Human/UC	Skin/rat skin burn model (in vivo)	Wnt4	EV-carried Wnt4 promoted β-catenin nuclear translocation and activity to enhance the proliferation and migration of skin cells	[21]
Human/AT	Brain/in vitro model of Alzheimer’s disease	Neprilysin/CD10	EV-mediated secretion and transfer of enzymatically active neprilysin led to the degradation of intracellular and extracellular Aβ in neuroblastoma cell lines	[22]
Human/BM	Immune system/human-into-mouse xenogeneic GVHD model (in vivo)	CD73	Inhibition of Th1 cell effector function via EV-CD73-mediated adenosine production, which activated the A2A receptor-mediated adenosine pathway in the Th1 cells	[23]

*Aβ* amyloid beta, *AT* adipose tissue, *BM* bone marrow, *ESC* embryonic stem cell, *EV* extracellular vesicle, *GVHD* graft-versus-host disease, *MIR* myocardial ischemia/reperfusion, *MSC* mesenchynal stem cell, *ND* not determined, *TGFβ* transforming growth factor beta, *Th1* T-helper type 1, *UC* umbilical cord

MSC-EVs primarily seem to support the proliferation and apoptosis avoidance of surviving tissue cells by modulating intracellular signaling pathways. The earliest studies found that the therapeutic effect of MSC-derived paracrine factors on myocardial ischemia/reperfusion injury (MIR) is largely ascribed to EV fractions [18, 19]. Although the responsible EV proteins had not yet been determined specifically, the subsequent study indicated that this therapeutic effect is mediated by restoring bioenergetics as evidenced by increased ATP and NADH levels, reduced oxidative stress via reduced c-JNK phosphorylation, and promoted proliferation via PI3K/Akt pathway activation [20]. Recently, Zhang et al. [21] elegantly demonstrated that MSC-EVs promoted recovery from skin burns by promoting skin cell proliferation. This proliferative effect was due to EV-Wnt4-mediated direct promotion of β-catenin nuclear translocation.

EVs allow MSC-derived membrane enzymes with therapeutic potential to have a unique functional mode. Our group reported that adipose tissue-derived mesenchymal stem cell (ADSC)-EVs contain enzymatically active neprilysin (also known as CD10), the rate-limiting amyloid beta (Aβ)-degrading enzyme in the brain [22]. Coculture experiments strongly suggested that ADSC-EVs were transferred to amyloid precursor protein-overexpressing Neuro-2a cells, thereby reducing both extracellular and intracellular Aβ levels. Currently, we are investigating the in vivo therapeutic potential of ADSC-EVs using animal models of Alzheimer’s disease. Another report also suggested the therapeutic potential of EV-associated enzymes in graft-versus-host disease (GVHD) [23]. Bone marrow (BM)-MSC-derived EVs carry enzymatically functional CD73 (also known as ecto-5′-nucleotidase), which metabolizes AMP into adenosine, a molecule that contributes to ATP signaling. Through this signaling, A2AR-expressing T-helper type 1 (Th1) cells are led to apoptosis.

## RNA-mediated therapeutic effects

One of the most attractive characteristics of EVs is their ability to transfer RNAs from one cell to another, thereby allowing the transferred RNAs to affect the recipient cells. Between 2006 and 2007, a sensational finding demonstrated that EV cargo mRNAs can be transferred and translated to proteins in recipient cells [24, 25]. Furthermore, in 2010 EVs were shown to transfer microRNAs (miRNAs) between cells, and the transferred miRNAs displayed RNA interference (RNAi) effects in the recipient cells [26–28]. In recent years, EV-containing RNAs have been shown to be transferred from MSCs to injured cells and to contribute to tissue recovery (Table 2).

**Table 2:** MSC-EV RNA-mediated therapeutic effects

Species/MSC origin	Injured organ/experimental model	Responsible RNA	Possible mechanisms for the therapeutic effect	Reference
Human/BM	Kidney/mouse model of AKI induced by glycerol (in vivo)	Predicted to be mRNAs	Induction of proliferation of surviving intrinsic epithelial cells	[29]
Human/BM	Kidney/mouse model of AKI induced by cisplatin (in vivo)	Predicted to be mRNAs	Induction of survival of tubular epithelial cells via anti-apoptotic effects	[30]
Human/BM	Kidney/rat model of AKI induced by ischemia/reperfusion (in vivo)	Predicted to be mRNAs	Proliferative and anti-apoptotic effects on surviving intrinsic epithelial cells	[31]
Human/BM	Lung/mouse endotoxin-induced or LPS-induced acute lung injury (in vivo)	KGF mRNA	Immunosuppressive effects partly through KGF elevation, which was caused by EV-mediated transfer of KGF mRNA	[32]
Rat/BM	Brain/rat model of middle cerebral artery occlusion (in vivo)	miR-133b	Induction of neurite outgrowth of neural cells	[33]
Rat/BM	Brain/rat model of middle cerebral artery occlusion (in vivo)	miR-133b	Promotion of functional recovery by increasing neuroblasts and induction of neurovascular plasticity by increasing vascular endothelial cells	[34]
Mouse/BM	Heart/mouse myocardial infarction model (in vivo)	miR-22	Reduction of apoptosis of ischemic cardiomyocytes by directly targeting methyl CpG binding protein 2 (Mecp2) via EV cargo miR-22	[35]
Rat/BM	Heart/rat regional MIR model (in vivo)	miRNA-19a	Reduction of the expression level of PTEN, a predicted target of miR-19a, thus activating the Akt and ERK signaling pathways	[37]

*AKI* acute kidney injury, *BM* bone marrow, *EV* extracellular vesicle, *KGF* keratinocyte growth factor, *LPS* lipopolysaccharide, *MIR* myocardial ischemia/reperfusion, *miRNA* microRNA, *MSC* mesenchynal stem cell, *PTEN* phosphatase and tensin homolog

Although more precise analyses are required, MSC-EV mRNAs are regarded as therapeutically beneficial in injured tissue recovery. The first evidence for the therapeutic effects of MSC-EVs was provided for kidney injury. Intriguingly, the therapeutic effects of MSC-EVs were partly mediated by the packaged mRNAs, which were suggested to be associated with proliferation, transcription regulation, and immunomodulation [29–31]. Another recent study demonstrated more specifically the association of the transfer of mRNA with acute lung injury (ALI) recovery [32]. EV-containing keratinocyte growth factor (KGF) mRNA was transferred from BM-MSCs to alveolar epithelial type II (ATII) cells and translated to protein. This elevation of KGF protein in ATII cells, in concert with the immunomodulatory effect of MSC-EVs, led to protective effects against ALI.

miRNAs are also proposed to be key molecules that are responsible for MSC-EV-mediated therapeutic potential. miR-133b, a regulator of tyrosine hydroxylase production and a dopamine transporter, is the best characterized MSC-EV miRNA with therapeutic effects on cerebral injury. This miRNA was transferred from BM-MSCs to injured neurons and then induced neurite outgrowth and promoted neural plasticity [33, 34]. Although several putative targets of miR-133b have been proposed, more detailed evidence is required to determine the critical role of MSC-EV miR-133b in the observed therapeutic effects. miR-22 in BM-MSC-EVs is reported to protect against ischemic heart disease by reducing cardiomyocyte apoptosis [35]. This anti-apoptotic effect of miR-22 was suggested to be due to direct targeting of methyl CpG binding protein 2 (mecp2), which is upregulated in the ischemic heart [36]. In addition, a similar therapeutic effect has been reported for miR-19a in BM-MSC-EVs, where its target is predicted to be phosphatase and tensin homolog (PTEN) deleted from chromosome 10 [37].

## Molecularly undefined mechanisms of MSC-EV-mediated therapeutic effects

Despite the rapidly increasing number of reports regarding the therapeutic effects of MSC-EVs, many lack detailed investigations identifying the molecule(s) responsible for these effects. As summarized in Table 3, the therapeutic effects of MSC-EVs include promotion of proliferation [38–42] and prevention of apoptosis [40, 42, 43] of surviving cells, modulation of the immune system [38, 41, 44–46], suppression of fibrosis [45, 47], and promotion of angiogenesis [41, 48, 49].

**Table 3:** MSC-EV-mediated therapeutic effects by undefined responsible molecules

Species/MSC origin	Injured organ/experimental model	Possible mechanisms for the therapeutic effect	Reference
Mouse/BM	Kidney/rat model of 5/6 subtotal nephrectomy (in vivo)	Fibrosis prevention, interstitial lymphocyte infiltrates, and absent tubular atrophy	[47]
Mouse/BM			
Human/UC	Lung/mouse hypoxic pulmonary hypertension model (in vivo)	Suppression of the hypoxic pulmonary influx of macrophages and the induction of anti-inflammatory and pro-proliferative mediators	[38]
Human/UC	Hind limb/rat ischemia model (in vivo)	Promotion of proliferation and tubular structure formation of endothelial cells in vitro and exemplified in vivo with the evidence of improvement in the blood flow recovery	[48]
Rat/BM	Brain/rat model of middle cerebral artery occlusion (in vivo)	Enhancement of neurite remodeling, neurogenesis and angiogenesis, which were evidenced by increases in axonal density and synaptophysin-positive areas, and doublecortin-positive neuroblast and endothelial cell numbers	[39]
Human/UC	Kidney/rat cisplatin-induced acute kidney injury model (in vivo) and in vitro culture of rat tubular epithelial cells exposed to cisplatin	Decrease in cisplatin-mediated renal oxidative stress and apoptosis in vivo and increase in renal epithelial cell proliferation in vitro	[43]
Human/ESCs	Immune system/mouse model of allogeneic skin graft (in vivo)	Induction of an attenuated proinflammatory cytokine response and enhanced anti-inflammatory cytokine production by monocytes, leading to polarized activation of CD4^+^ T cells to CD4^+^CD25^+^FoxP3^+^ regulatory T cells (Tregs)	[46]
Human/ESCs	Liver/CCl_4_-induced mouse liver injury model (in vivo)	Upregulation of the priming-phase genes during liver regeneration, which subsequently led to increased expression of proliferation-related proteins, PCNA and cyclin D_1_	[40]
Horse/AT	Vascular system/ex vivo rat aortic ring and in vitro scratch assays	Mechanisms have not been identified	[49]
Human/BM	Lung/SiO_2_-induced mouse idiopathic pulmonary fibrosis model (in vivo)	Downregulation of inflammatory response and suppression of fibrosis as evidenced by decreased collagen deposition	[45]
Human/AT	Immune system/in vitro culture of stimulated T cells	Inhibitory effect on the differentiation and activation of T cells and reduced T cell proliferation and IFNγ release in in vitro stimulated cells	[44]
Rat/BM	Brain/rat cortical impact rat model of traumatic brain injury (in vivo)	Increased endothelial cells in the lesion boundary zone and dentate gyrus, increased numbers of newly formed immature and mature neurons in the dentate gyrus and reduced neuroinflammation	[41]
Mouse/AT	Brain/in vitro culture of neuronal cells exposed to oxidative stress and ex vivo cerebellar slice cultures treated with lysophosphatidylcholine	Protection of neurons from apoptotic cell death, promotion of remyelination and activation of nestin-positive oligodendroglial precursors	[42]

*AT* adipose tissue, *BM* bone marrow, *ESC* embryonic stem cell, *EV* extracellular vesicle, *IFNγ* interferon gamma, *MSC* mesenchynal stem cell, *UC* umbilical cord

Because the primary focus of this article is the molecular mechanisms underlying the therapeutic effects of MSC-EVs from the point of view of their content molecules, we do not provide a detailed review of those reports that have not elucidated the responsible molecules. Nevertheless, considering that these reports provide much insight, we will highlight some of them here. In a mouse model of allogeneic skin grafts, MSC-EVs contributed to immunosuppression by increasing the number of CD4^+^CD25^+^FoxP3^+^ regulatory T cells (Tregs) [46]. This immunosuppressive effect is distinct from that of MSC-EVs observed in GVHD, where MSC-EVs did not affect the number of Tregs but directly decreased the numbers of Th1 cells and cytotoxic T cells [23]. These two reports suggest that even phenotypically similar outputs elicited by MSC-EVs are likely to be context dependent. Thus, clarifying the difference between these two distinct immunosuppressive effects is intriguing. To this end, identifying the EV molecule(s) responsible for these effects more precisely will be important. Another interesting report suggested the surprising capacity of EVs as a transportation tool between cells. Islam et al. [50] reported that MSCs protect against ALI, and these authors ascribe this therapeutic effect to EV-mediated intercellular transfer of mitochondria. Before this report, lung epithelial cells harboring mitochondrial injury were cocultured with MSCs and were reported to receive mitochondria from MSCs, thereby recovering their proliferative capacity and lung functions [51]. Islam et al. [50] claim that this mitochondrial transfer from MSCs to lung epithelial cells is mediated by EVs; however, no direct evidence exists showing that the addition of MSC-EVs to cultured epithelial cells resulted in mitochondrial transfer. In contrast, another group [32] identified mitochondrial genes in MSC-EVs, implying the involvement of mitochondrial transfer in the therapeutic outcome of MSC-EVs. Further investigation regarding this possibility is required. It should also be noted that the stress of pathophysiological conditions impacts the effects of MSC-EVs. For example, hypoxia, ischemic conditioning, or inflammatory conditioning of MSCs is shown to regulate protein or miRNA packaging into EVs and to affect their functional properties [33, 52, 53].

## Future perspectives

In the next few years, exploring and understanding more comprehensively the therapeutic effects of MSC-EVs by taking advantage of omics data will be important. Specifically, we can expect to be able to predict the beneficial characteristics of MSC-EVs. An earlier study by Kim et al. [54] performed proteome analysis of MSC-EVs and proposed several candidate signaling pathways that were expected to be activated by MSC-EVs. These predicted pathways included the Wnt, transforming growth factor beta (TGFβ), mitogen-activated protein kinase (MAPK), peroxisome proliferator-activated receptor (PPAR), and bone morphogenetic protein (BMP) signaling pathways. Of these pathways, the MAPK and Wnt signaling pathways have been confirmed to be activated following MSC-EV administration [20, 21, 37, 40, 43]. In addition to pathway prediction, predicting a single EV molecule as a therapeutic effector will also be beneficial. Although not predicted as candidate therapeutic effectors, neprilysin/CD10 and CD73 were identified in the proteomic analyses performed by Kim et al. [54]. These two molecules were later proposed to be therapeutically valid, as already reviewed [22, 23]. Another recent study performed RNA sequencing (RNA-seq) of MSC-EVs [55]. According to this study, EVs were preferentially rich in mRNAs for transcription factors and angiogenesis-associated genes. Of these transcription factors, FoxP3 may contribute to immunosuppressive effects because FoxP3 is a master gene for Treg lineage specification. In contrast, intriguingly, RNA-seq generated reads for at least 386 annotated miRNAs but only four of these were enriched in EVs compared with the original MSCs. This finding suggests that other mechanisms may underlie the therapeutic effects of MSC-EVs. Indeed, a more recent RNA-seq study also questioned the presently well-accepted hypothesis that miRNAs are the primary effectors of the therapeutic potential of MSC-EVs. Baglio et al. [56] performed RNA-seq to characterize the full small RNAome of MSC-EVs. Their data indicate that miRNAs and small nucleolar RNAs (snoRNAs) are significantly enriched in cells, while tRNAs and repeats form a defined pool of RNAs heavily enriched in exosomes. These authors also found that tRNA halves, which are 30–40 nucleotides long and which are produced by the cleavage of mature cytoplasmic tRNAs, appear to be massively sorted into MSC-EVs. tRNA halves have emerged as a novel class of small noncoding RNAs that might have biological functions [57]; in particular, 5′ tRNA halves are suggested to serve as translation suppressors [58, 59]. In addition, 5′ tRNA halves are shown to be present in immune cell-derived EVs [60] and in body fluids such as blood [61, 62] and semen [63], suggesting their potential biological significance. In this regard, the study by Baglio et al. noted the importance of investigating not only miRNAs but also tRNAs to further explore and understand MSC-EV-mediated therapeutic effects.

Second, we would like to emphasize the possible oncogenic risks entailed by MSC-EVs. The pro-proliferative effects of MSC-EVs on injured cells imply the possibility that MSC-EVs accelerate cancer progression. At present, whether MSC-EVs have pro-cancer or anti-cancer effects remains controversial. Some studies have claimed that MSC-EVs support cancer progression [64–68], whereas others have shown the anti-tumorigenic effects of MSC-EVs [69–74]. Although we do not have a reasonable explanation at present, two reports by Zhu et al. [68, 72] have provided insight regarding this controversy. The authors investigated the effects of MSC-EVs on two different types of cancer. While MSC-EVs attenuated the growth of bladder cancer cells [72], MSC-EVs promoted the growth and aggressiveness of renal cancer cells [68]. These contradictory observations suggest that MSC-EVs affect cancer cells in a cancer type-dependent manner. In addition, considering the notion of cancer recurrence, apparent anti-cancer effects may reflect the early stage of cancer cell dormancy. Indeed, some studies have suggested that anti-proliferative effects on cancer cells should be regarded as induction of cancer cell dormancy, thereby providing a platform for cancer recurrence [75, 76]. More comprehensive insight into this controversial issue is a prerequisite for applying MSC-EVs to clinical settings.

Third, we should account for an issue regarding inter-individual variability in MSC-EV function. MSCs that are indistinguishable based on marker characterization could display strongly different capacities to produce cytokines and respond to inflammatory licensing [77]. Donor age and gender also affect characteristics of human MSCs, such as surface marker profiles and clonogenic capacity [78]. At present, there is no report on the inter-individual variability of MSC-EVs, and thus we should comprehensively interrogate this issue and explore criteria for clinical use of MSC-EVs. For this purpose, it will be important to know the relationship between the molecular signatures of MSC-EVs and their therapeutic efficacy, and also to know the relationship between donor MSC characteristics and the functionality of the EVs secreted by them. It might also be important to know whether there is any relationship between donor MSC characteristics and their productivity of EVs. Such information will help us to predict clinical outcomes from administration of a patient’s derived MSC-EVs.

## Conclusion

MSC-EVs have beneficial effects on recovery from a variety of tissue injuries. These effects are mediated by MSC-EV content molecules including proteins and RNAs, and elucidation of the underlying mechanisms of these effects is now in progress. In addition, recently available omics data suggest a possibility to further explore and understand the key molecular basis of these beneficial effects of MSC-EVs. For the realization of clinical applications of MSC-EVs, however, we should be aware of the oncogenic risks that may be associated with MSC-EVs.

## Note

This article is part of a thematic series on *Extracellular vesicles and regenerative medicine* edited by Jeffrey Karp, Kelvin Ng and Armand Keating. Other articles in this series can be found at http://stemcellres.com/series/EVRM.

## Abbreviations

ADSC: Adipose tissue-derived mesenchymal stem cell
ALI: Acute lung injury
ATII: Alveolar epithelial type II
Aβ: amyloid beta
BM: Bone marrow
BMP: Bone morphogenetic protein
EV: Extracellular vesicle
GVHD: Graft-versus-host disease
KGF: Keratinocyte growth factor
MAPK: Mitogen-activated protein kinase
MIR: Myocardial ischemia/reperfusion injury
miRNA: microRNA
MSC: Mesenchymal stem cell
MSC-EV: Extracellular vesicle derived from mesenchymal stem cell
PPAR: Peroxisome proliferator-activated receptor
PTEN: Phosphatase and tensin homolog
RNAi: RNA interference
RNA-seq: RNA sequencing
TGFβ: Transforming growth factor beta
Th1: T-helper type 1
Treg: Regulatory T cell
